# Habitat suitability mapping of the black coral *Leiopathes glaberrima* to support conservation of vulnerable marine ecosystems

**DOI:** 10.1038/s41598-021-95256-4

**Published:** 2021-08-02

**Authors:** V. Lauria, D. Massi, F. Fiorentino, G. Milisenda, T. Cillari

**Affiliations:** 1 IRBIM - Institute for Marine Biological Resources and Biotechnologies, National Research Council CNR (CNR), Mazara del Vallo, TP Italy; 2 Zoological Station Anton Dohrn, Lungomare Cristoforo Colombo Complesso Roosevelt, 90149 Palermo, Italy; 3 Institute for Environmental Protection and Research (ISPRA), Lungomare Cristoforo Colombo Complesso Roosevelt, 90149 Palermo, Italy

**Keywords:** Ecology, Biodiversity, Ecological modelling

## Abstract

The black coral *Leiopathes glaberrima* is an important habitat forming species that supports benthic biodiversity. Due to its high sensitivity to fishing activities, it has been classified as indicator of Vulnerable Marine Ecosystems (VMEs). However, the information on its habitat selection and large-scale spatial distribution in the Mediterranean Sea is poor. In this study a thorough literature review on the occurrence of *L. glaberrima* across the Mediterranean Sea was undertaken. Predictive modelling was carried out to produce the first continuous map of *L. glaberrima* suitable habitat in the central sector of the Mediterranean Sea. MaxEnt modeling was used to predict *L. glaberrima* probability of presence as a function of seven environmental predictors (bathymetry, slope, aspect North–South and East–West, kinetic energy due to currents at the seabed, seabed habitat types and sea bottom temperature). Our results show that bathymetry, slope and aspect are the most important factors driving *L. glaberrima* spatial distribution, while in less extent the other environmental variables. This study adds relevant information on the spatial distribution of vulnerable deep water corals in relation to the environmental factors in the Mediterranean Sea. It provides an important background for marine spatial planning especially for prioritizing areas for the conservation of VMEs.

## Introduction

*Leiopathes glaberrima* (Esper, 1788) is a black coral species belonging to the family Leiopathidae, it is considered among the most important components of deep corals community of the rocky substrata on the platform and slope in the Mediterranean Sea^1,2^. This species has a broad spatial distribution, occurring from the Pacific Ocean to the northeast Atlantic Ocean and Mediterranean Sea^3^, and a wide bathymetric range (between 100 and 2048 m^4^) but in the Mediterranean Sea is mainly found at depths ranging from 200 to 500 m^5^. *L. glaberrima* is a tall, arborescent species characterized by a branched canopy that may exceed 2 m in height^6,7^. In the Mediterranean Sea this species is present in several Cold-Water Corals (CWC) provinces (e.g. northern Ionian Sea, southern Adriatic Sea, Strait of Sicily, eastern Alborán Sea) often associated to other hard bottom deep water corals such as *Madrepora oculata* and *Lophelia pertusa*^6^.

It provides essential habitat for several marine species and increases benthic biodiversity^8^. For example, some elasmobranch species such as the lesser spotted dogfish (*Scyliorhinus canicula*) attaches its egg cases on *L. glaberrima* branches^5,8^. In addition, as many other habitat forming species it plays a key role in the maintenance of ecosystem functioning^9^. This black coral is one of the longest living marine organisms that can reach millennial ages ranging between 483 and 4265 years^10^. In the Mediterranean Sea two colonies were dated of approximately 2000 years off Carloforte (southwest coast of Sardinia^7^) and 650 years off Malta Island (Strait of Sicily^11^) by using radiocarbon (14C). As other suspension feeder organisms (i.e. their diet is mainly formed of zooplankton and particulate organic matter^7,12,13^) *L. glaberrima* habitat selection is strongly influenced by the hydrodynamic regime and type/availability of substrata^13,14^. Indeed, several colonies are found on exposed hard bottoms, especially on ridges and flanks of canyons or seamounts, bench terraces with low silting^7,15^ or white coral mounds^16^. Usually, colonies have a fragmented spatial distribution, typical of all arborescent anthozoans characterized by a limited larval dispersion^17–19^. Patches show a high density of colonies, such that the occurrence of few sparse colonies with traces of damage has been considered as a bioindicator of impacted site^20^. Despite some information about its spatial distribution is available in other areas (Gulf of Mexico)^10^ little is known on this species large-scale spatial distribution and habitat preference in the Mediterranean Sea.

*Leiopathes glaberrima* as other CWC, is highly vulnerable to human induced impacts (e.g. bottom fishing, petroleum exploitation, pollution, seabed mining and ocean acidification) and now recognized as endangered by IUCN^5^. Fishing is one of the most damaging activity on its populations, as this black coral has been subjected in the past to collection for jewelry trade^21,22^. It has been reported that about 100–150 kg y^−1^ of black coral presumably belonging to *L. glaberrima* was caught in the Strait of Sicily (close to Malta Island) from 1984 to 1987^21^. However, the strongest impact is due to the indirect effects of fishing activities as this species is by-catch of bottom trawlers and longliners^20,23^. In particular trawlers reduce this black coral coverage on the swept bottoms, while longliners damage colonies because of the abrasion by entangled gears^20^. In addition, as of its life history traits (i.e. long-life span, slow grow rates, long reproductive cycle and low recruitments) and sensitivity to fishing activities, since 2013 *L. glaberrima* has been listed also in the Annex II of the SPA/BD protocol of the Barcelona Convention. For these reasons, a comprehensive spatial mapping of its distribution across the Mediterranean Sea is required so that management bodies can enhance appropriate conservation measures^21^, furthermore *L. glaberrima* has been listed as indicator of Vulnerable Marine Ecosystems (VMEs)^24^. Protecting VMEs is a legal obligation for Regional Fishery Management Organizations since 2008 (under the United Nations General Assembly Resolutions 59/25, 61/105 and 64/72), in addition the need for a sustainable management of deep-sea fisheries, while conserving VMEs, has been highlighted with several proposals by GFCM in 2017. These included both the adoption of environmental and biological indicators to identify VMEs location, and the establishment of an “encounter protocol” to avoid fishing on these vulnerable habitats. Within this framework, knowledge on areas where *L. glaberrima* and other VMEs indicators are concentrated should be the basis for a spatial approach to fishery management that accounts for VMEs protection. In this direction, the adoption of new technologies (e.g. multibeam, echosounder, Remotely Operated Vehicle) can be helpful to increase the efficiency of seafloor mapping^7,25^.

Species Distribution Models (SDMs) relate the environmental conditions of sites where the species has been observed to the conditions of the study area to identify locations where species are likely to occur^26^ so that conservation efforts can target specific areas. Also, the model outputs are extremely useful to understand the factors influencing spatial patterns and species habitat preference^27^. These models have been largely used to identify the main ecological factors influencing CWC distribution at global scale^10,28–31^.

Recently, an updated synopsis of *L. glaberrima* distribution in the Mediterranean Sea highlighted several distribution hotspots (i.e. Carloforte Island in the Sardinian Sea, the Marco Bank and off the Pontine Islands in the Southern Tyrrhenian Sea, and the Malta Graben in the Strait of Sicily) which have been proposed for implementation of Fishery Restricted Areas^32^. Considering the growing concern on the protection of VMEs and the need to identifying conservation areas that can inform spatial management plans, it is necessary to have a thorough knowledge on the habitat suitability of this species in the Mediterranean Sea.

The aims of this study are: (1) to create the first continuous map of *L. glaberrima* suitable habitat in the Mediterranean Sea; (2) to understand the role of environmental factors on this species habitat selection. Maximum Entropy (MaxEnt) habitat suitability model was used to model species occurrence data as a function of environmental descriptors (i.e. depth, slope, aspect North–South and East–West, seabed habitat type, kinetic energy due to the currents at the bottom and sea bottom temperature). Successively predictive distribution map was produced to identify species-specific spatial pattern. This study adds important knowledge on the habitat preference of a vulnerable coral species and can be used for the development of marine spatial planning initiatives including networks of Fishery Restricted Areas aimed to the protection of VMEs.

## Material and methods

### Study area

**Figure 1:**
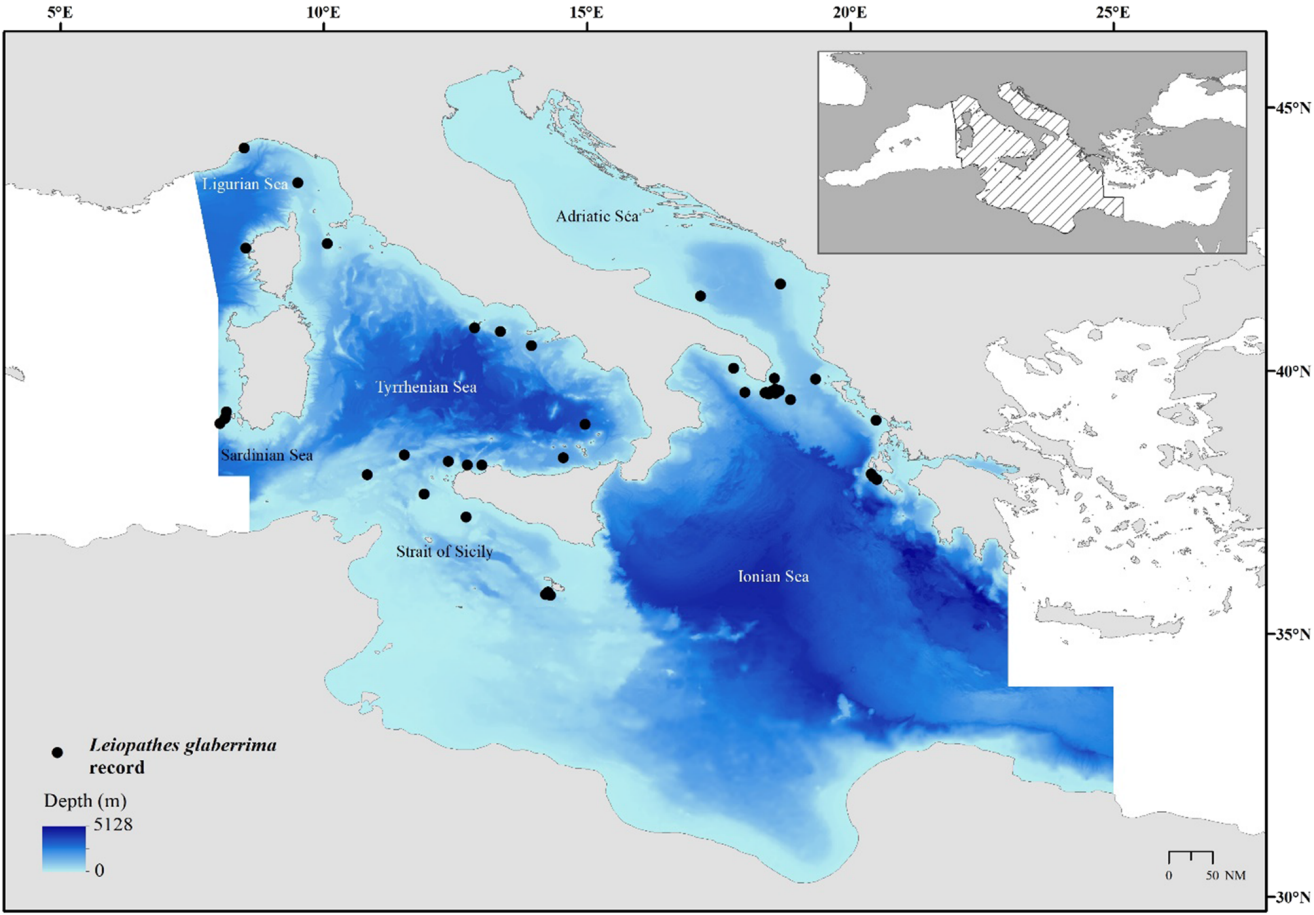
Study area and occurrences of *Leiopathes glaberrima* (black dots) collated from literature review. The bathymetric map is also showed. This map was created with ArcGIS version 10.3 http://www.esriitalia.it by Tiziana Cillari.

Our study area is the central sector of Mediterranean Sea, since the records of *L. glaberrima* reported in literature are more numerous here than in the western and eastern Mediterranean sectors. This area includes the Sardinian Sea, the Ligurian Sea, the Tyrrhenian Sea, the Strait of Sicily, the Ionian Sea, and the Adriatic Sea (Fig. 1). The central sector of the Mediterranean Sea is characterised by complex seafloor morphology and hydrodynamic processes^33,34^, with a wide range of depths, including shallow banks for example in the western part of the Strait of Sicily (about 100 m) and deeper areas in the Tyrrhenian Sea and Ionian Sea reaching depths of about 3800 and 5300 m, respectively (Fig. 1). This area is particularly important in terms of water masses circulation, representing a crossroads of different circulation belts occurring in the Mediterranean Sea. One of the main is represented by the inflow of the Atlantic Water stream (AW), flowing in the upper 50–100 m layer and coming from the Gibraltar Strait, which is transformed into Levantine Intermediate Water (LIW) in the Eastern part of the Mediterranean basin (flowing in the layer 200–600 m towards the Gibraltar Strait and the Atlantic Ocean). The deep-water masses are distinct between the western and eastern parts of the basin since the Strait of Sicily sill has a maximum depth of 500 m. The Western Mediterranean Deep Waters (WMDW) and the Eastern Mediterranean Deep Waters (EMDW) are formed in the Gulf of Lion area (southeast of France) and the southern Adriatic Sea, the Rhodes gyre and in the Sea of Crete, respectively^35^. However, the LIW are also influenced by components of the deep waters coming from the Adriatic Sea and Gulf of Lion^34^. At smaller scale the Sardinia Channel, together with the Strait of Sicily and the Sardinia-Sicily passage, represent crucial areas in the Mediterranean Sea in the control of water mass exchange between the Eastern and the Western Mediterranean^36^. The hydrology of the central sector of the Mediterranean Sea is particularly important for CWC as several studies have showed the importance of modified LIW on their habitat selection and distribution^6,37^.

### Species presence data and environmental variables

The presence data points of *L. glaberrima* were extracted from a synoptic review of this species distribution in the Mediterranean Sea^32^, updated with other records reported in more recent literature as showed in the Supplementary material (Table S1). When the precise coordinates were not available, these were estimated from detailed indication of the finding area based on locality and depth description. A total of 45 finding sites were identified as showed in Fig. 1.

**Figure 2:**
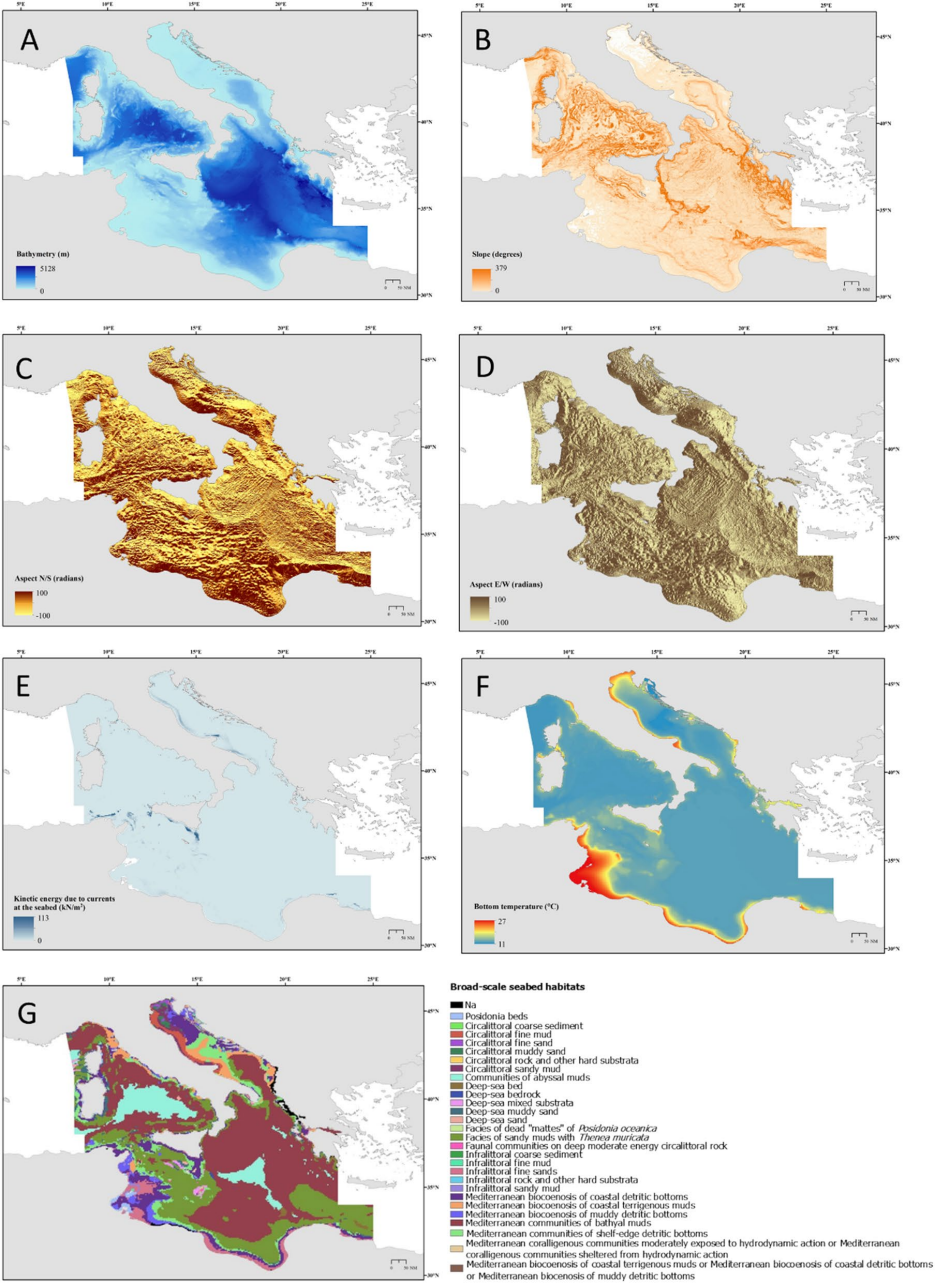
The spatial patterns of the environmental variables used in the MaxEnt model. These include (**A**) depth (m); (**B**) slope (degrees) values range from to 0° to 90° with low slope values corresponding to flat terrain and higher values to steeper terrain; (**C**) aspect north–south and east–west (**D**) scaled to 100 (radians); (**E**) Kinetic Energy due to currents (kN/m^2^); (**F**) sea bottom temperature (°C); (**G**) broad scale seabed habitats. These maps were created with ArcGIS version 10.3 http://www.esriitalia.it by Tiziana Cillari.

For modelling construction seven environmental variables were used (i.e. depth, slope, aspect North–South and East–West, seabed habitat types, kinetic energy due to currents at the bottom as proxy of current intensity, and sea bottom temperature) as shown in Fig. 2 and Table 1. Depth (Fig. 2A) is the major environmental gradient that influences species spatial distribution, and in the case of corals, it has been shown to be a key factor driving their patterns^29,31^. For this study bathymetry data were extracted from EMODnet database (EMODnet Digital Bathymetry, DTM 2016)^38^, a European marine dataset developed for spatial ecology.

**Table 1:** List of environmental predictors, and relative sources, used in the habitat suitability model of *Leiopathes glaberrima* in the central sector of the Mediterranean Sea.

Environmental variable	Data source	Reference period	Resolution (Degree)	Note
Bathymetry (m)	EMODnet Bathymetry Consortium	2016	0.002*0.002	
Bathymetric slope (degrees)	MARSPEC database	2009	0.008*0.008	A scaling factor of × 10 has been applied
Aspect N/S and E/W (radians)	MARSPEC database	2009	0.166*0.166	A scaling factor of × 100 has been applied
Kinetic energy due to currents at the seabed (kN/m^2^)	EMODnet Seabed Habitats initiative	2016–2018	0.041*0.041	
Bottom temperature (°C)	Copernicus Marine Service	2018	0.041*0.041	
Broad-scale seabed habitats (EUNIS classification)	EMODnet Seabed Habitats initiative	2019	240*240	

Slope and aspect are largely used in SDMs as indices of the seabed morphology, these parameters are frequently applied to approximate the physical processes that can interact between current and topography^39,40^.

Slope describes the rate of change in elevation over distance; values range from to 0° to 90° with low values of slope are associated with flat ocean bottoms or areas of sediment deposition, while higher values indicate potential rocky ledges. Recent studies on the spatial distribution of *L. glaberrima* indicates the preference of this species for rocky substrata and bench terraces with low silting^7,15,23^.

Aspect identifies the orientation of the seabed at any given location and provides information on the exposure of an area to local and regional currents^41^. This seabed feature can affect the currents regimes and therefore the flux of suspended food material on the sea bottom^42^. Aspect has been used in other studies of habitat suitability of CWC as it represents a proxy for other ecological processes, such as food availability^43^. It is measured in radians (ranging from − 1 to + 1) and divided into two components: North–South and East–West gradient that describe the direction that the surface slope faces. For this study, both slope and aspect were collected from the MARSPEC database^44^(Fig. 2B–D; Table 1).

As proxy for currents intensity, we used the velocity of kinetic energy at the seabed, this environmental predictor was created by the EMODnet Seabed Habitats project consortium using E.U. Copernicus Marine Service Information (available at https://www.emodnet-seabedhabitats.eu/access-data/download-data/?linkid=mediterranean_KE_currents) (Fig. 2E and Table 1). Bottom currents are important drivers for the habitat selection of deep-sea corals as they influence food supply and larval dispersal^37,45,46^.

Sea bottom temperature data (Fig. 2F) were collected from the Copernicus Marine Service^47^. Sea temperature is an important environmental factor that drives the habitat selection of deep-sea corals, it can affect their calcification rate, physiology, and biochemistry^48^.

Finally, the broad scale seabed habitat type was also used as predictor of *L. glaberrima* spatial distribution (Fig. 2G and Table 1). Data came from the EUNIS database, a comprehensive pan-European system for habitat identification available at http://www.emodnet-seabedhabitats.eu/. The classification is hierarchical and covers all seabed types from natural to artificial, from terrestrial to freshwater and marine.

### Modelling approach

Priori model fitting, all explanatory variables were tested for collinearity using the Variance Inflation Factor^49^. Maximum Entropy method (MaxEnt) (software version 3.4.1^50^ downloaded from https://biodiversityinformatics.amnh.org/open_source/maxent/) was used to predict the potential distribution of *L. glaberrima* as a function of seven environmental predictors. MaxEnt is a well-known machine-learning modelling approach largely used to model species geographic distributions especially in cases of presence-only data (for example museum collections^51–52^). This approach is particularly suitable even with small sample sizes^53,54^. It has been frequently used for predictive CWC habitat mapping in several marine ecosystems^10,29,55,56^.

The algorithm used in MaxEnt aims to find the largest spread, or maximum entropy, in the geographic dataset composed of occurrence records of *L. glaberrima*, in relation to the environmental predictors. MaxEnt starts with a uniform distribution of occurrence probability values for *L. glaberrima* over the study area and conducts an optimization routine that iteratively improved model fit, measured as the loss of entropy (i.e. the “gain” of information). The model output is a probability map of species distribution that varies from 0 (lowest probability or least suitable habitat) to 1 (high probability or most suitable habitat)^50,51^.

Before running the MaxEnt model, all environmental variables were converted to digital continuous maps (.asc files) with the same geographic extent, bounds and cell size (resolution). A cell size of 200 m × 200 m was selected. Model settings were chosen according to the similar studies on corals^55,56^. Some default settings were left unchanged (convergence threshold of 10^−5^, maximum iterations 500, maximum background points 10,000) that proved efficient in many studies (see Bargain et al. 2017^55^ and reference there in). A regularization multiplier of 3 and a default prevalence of 0.7 was set respectively to reduce the over-fitting and obtain higher habitat suitability values in known areas of coral occurrences^55,56^. The feature class (transformation of the environmental variables expressing the constraints) "Hinge only" was selected to improve model performance when the number of presence records is at least 15^57,58^. Finally, the logistic outputs, that gives an estimate of probability of presence conditioned on environmental variables and easier to interpret^59^ was selected.

The MaxEnt model was used with a k-fold cross-validation procedure to assess the uncertainty in predictions of the model. Presence data were splitted into 10 randomly generated partitions to compare the training datasets and testing datasets for model validation. This allowed obtaining out-of-sample estimates of predictive performance and estimates of uncertainty around fitted functions^58^. The predictive accuracy of the model was calculated by ROC (Receiver Operating Characteristics) analysis through comparison of the Area Under the ROC Curves (AUC)^51^. The AUC-value, ranging from 0 to 1, indicates how well the model fits the data: a test AUC below 0.5 means that the model is no better than random, an AUC of 1 means the model is ideal, whereas an AUC higher than 0.7 can be considered as appropriate^55,60^. Test gain (which is a measure of goodness of fit) was also applied to evaluate how close the model is to the test presence samples (if the gain is 2, it means that the average likelihood of the presence samples is exp(2) = 7.4 times higher than that of a random background pixel^61^.

Finally, the contribution of each variable to the predictive model was also examined. Jackknife plots and variable response curves were selected to assess the importance of each variable. Jackknife tests compare the predictive performance of the model with only one of each variable and then with all the variables except the variable tested first. The mean model of 10 replicates was then used for habitat suitability mapping. The obtained probability map of *L. glaberrima* distribution was processed in ArcGIS 10.3 software to display the probability of presence in the study area.

## Results

### Modelling evaluation

**Figure 3:**
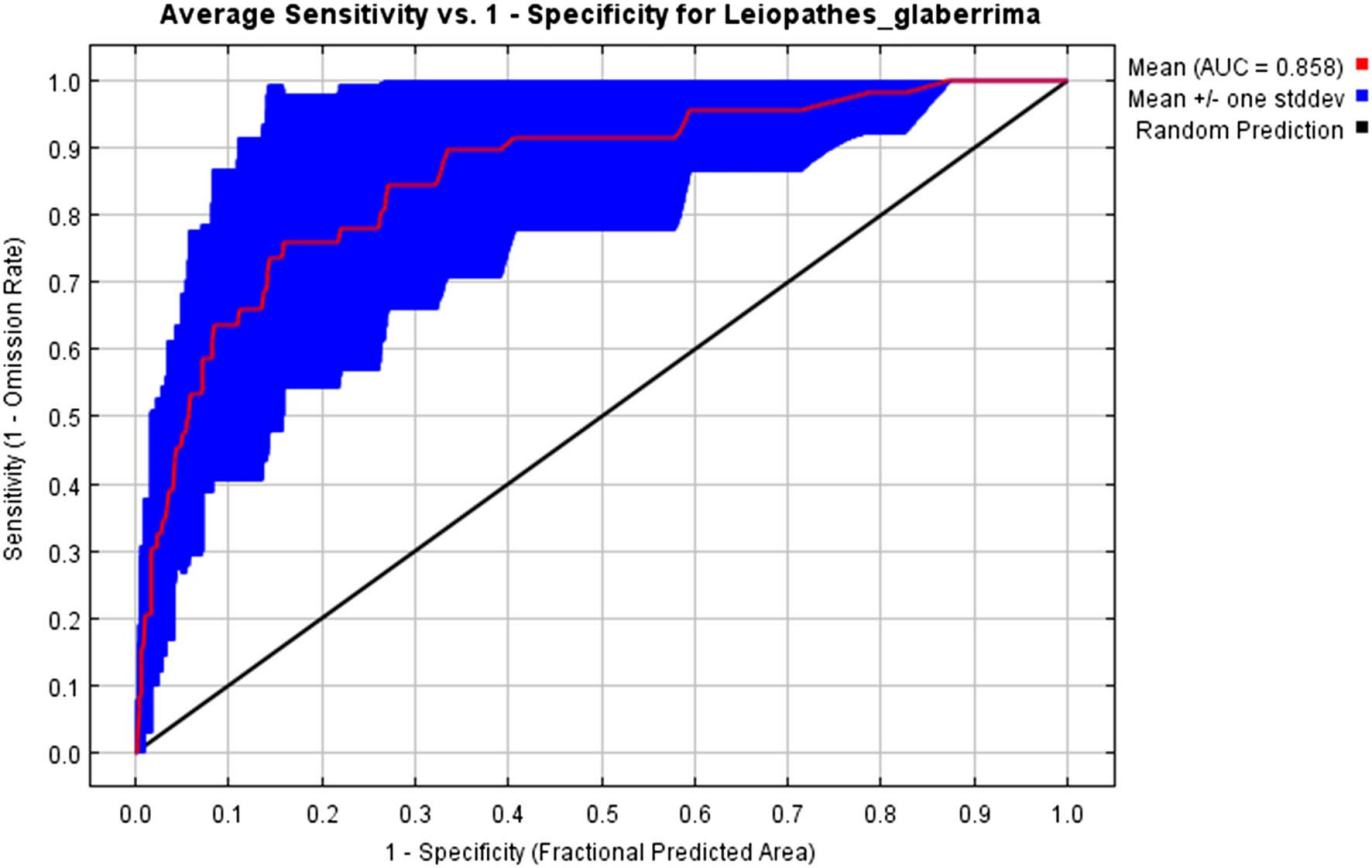
ROC (Receiver Operating Characteristic) curves for the training dataset of *Leiopathes glaberrima*. AUC: Area Under the Curve.

The MaxEnt model performed well, the mean AUC value of the ten replicates was 0.858 and a standard deviation of 0.101 (Fig. 3; Table 2) showing that our model was significantly better than random. The measure of goodness of fit obtained with our mean model and test gain with all variables of 0.97, meaning that the average likelihood of the presence samples was 2.64 [exp(0.97)] times higher than that of a random background pixel. This high AUC supported by a moderate-test gain indicates that the model built for the central sector of the Mediterranean Sea was powerful for predicting *L. glaberrima* habitat distribution.

**Table 2:** Relative contribution of each environmental predictor variable to the *Leiopathes glaberrima* distribution model.

Variable	Percent contribution
Bathymetry	45.3
Slope	21.6
Aspect E/W	12.1
Kinetic energy due to currents at the seabed	8.1
Broad-scale seabed habitats	7.3
Aspect N/S	4.9
Bottom temperature	0.8

### Assessment of variable importance within the model

All the environmental predictors were not autocorrelated (VIF < 2), therefore utilized for model construction. A total of 45 presence points was used to model *L. glaberrima* occurrence in the central sector of the Mediterranean Sea. The final model retained all seven environmental variables, and the average training AUC for the replicate runs was 0.858 (Fig. 3; Table 2). Bathymetry, slope, aspect EW, and kinetic energy due to currents at the seabed were the main contributors to the model with a combined contribution of 87.1% (Table 2), whilst the remaining three predictors (i.e. broad-scale seabed habitats, aspect NS and sea bottom temperature) contributed for 12.9%.

**Figure 4:**
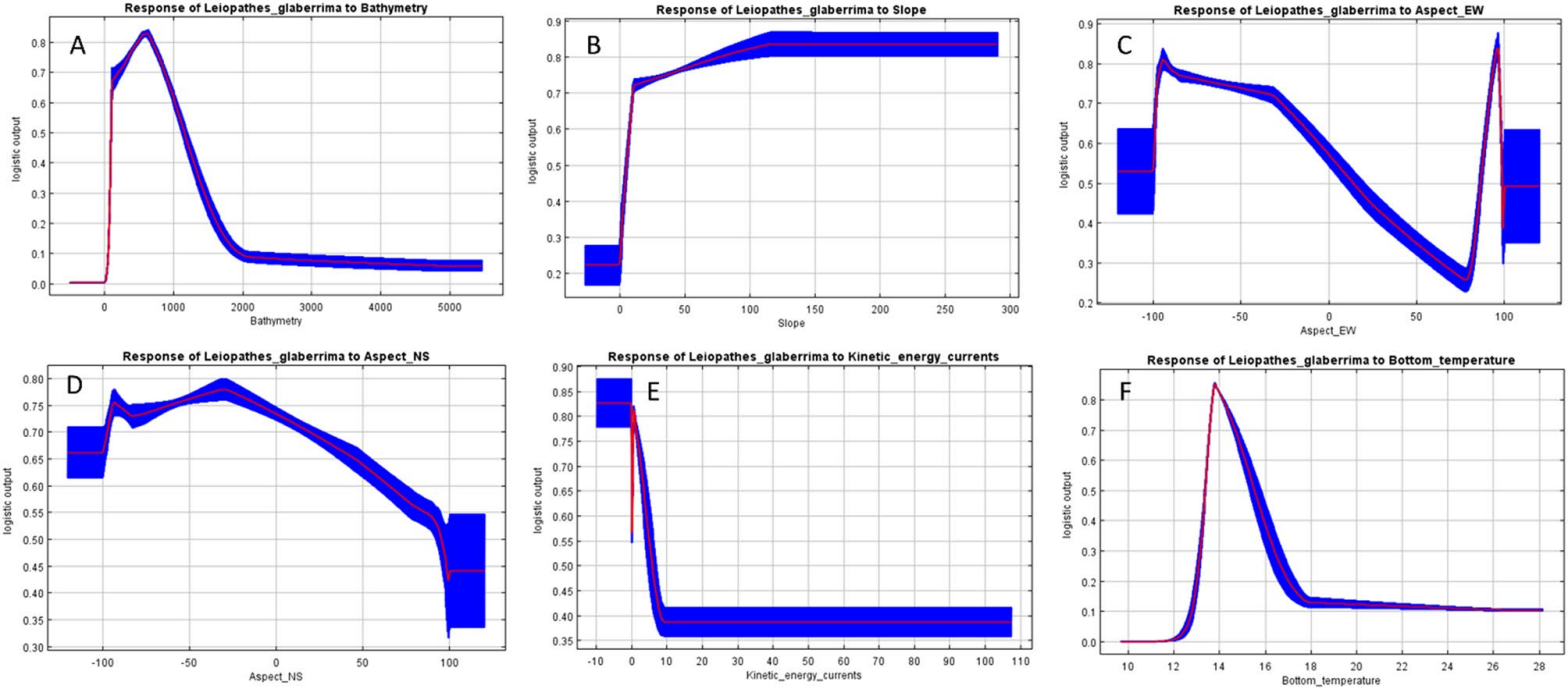
Response curves of the predicted probability of *Leiopathes glaberrima* presence in relation to the continuous environmental variables in our study area. The curves show the mean response of the 10 replicate MaxEnt runs (red) + /-standard deviation (blue).

**Figure 5:**
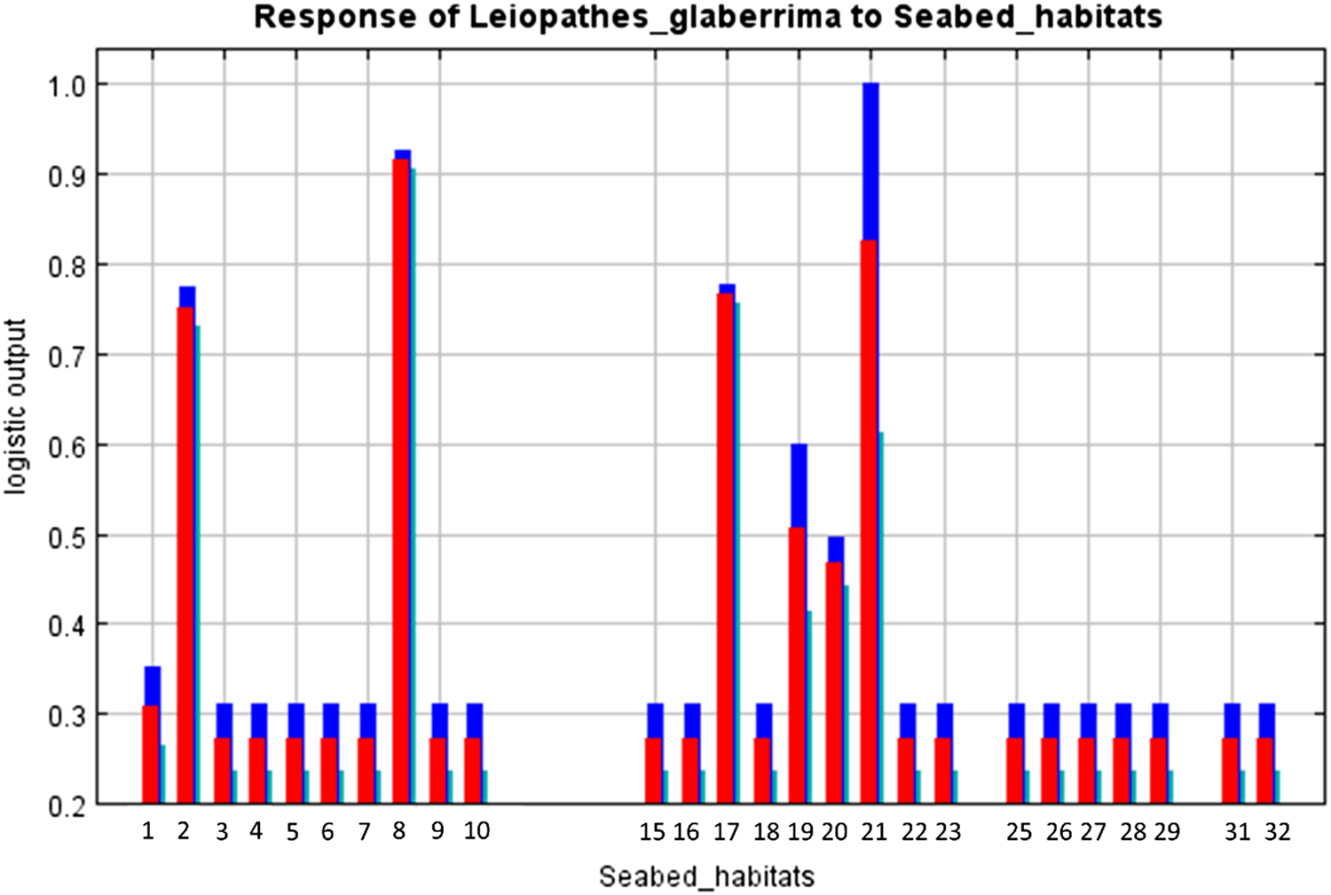
Histograms of the probability of *Leiopathes glaberrima* occurrence for category of broad-scale seabed habitat type in our study area (number in x-axis). The bars show the mean response of the 10 replicate MaxEnt runs (red) + /-standard deviation (blue). Category of seabed habitat (from EUNIS classification): (1) Mediterranean biocoenosis of coastal detritic bottoms; (2) Mediterranean communities of shelf-edge detritic bottoms; (3) Deep-sea mixed substrata; (4) Faunal communities on deep moderate energy circalittoral rock; (5) Deep-sea bedrock; (6) Mediterranean coralligenous communities moderately exposed to hydrodynamic action or Mediterranean coralligenous communities sheltered from hydrodynamic action; (7) Infralittoral fine sands; (8) Deep-sea muddy sand; (9) Infralittoral rock and other hard substrata; (10) Deep-sea sand; (11) Infralittoral coarse sediment; (13) Mediterranean biocoenosis of coastal terrigenous muds or Mediterranean biocoenosis of coastal detritic bottoms or Mediterranean biocenosis of muddy detritic bottoms; (15) Infralittoral sandy mud; (16) Mediterranean biocoenosis of muddy detritic bottoms; (17) Facies of sandy muds with Thenea muricata; (18) Infralittoral fine mud; (19) Mediterranean biocoenosis of coastal terrigenous muds; (20) Mediterranean communities of bathyal muds; (21) Posidonia beds; (22) Communities of abyssal muds; (23) Facies of dead "mattes" of Posidonia oceanica; (25) Na; (26) Deep-sea bed; (27) Circalittoral sandy mud; (28) Circalittoral fine mud; (29) Circalittoral muddy sand; (30) Circalittoral coarse sediment; (31) Circalittoral rock and other hard substrata; (32) Circalittoral fine sand.

The average response of *L. glaberrima* presence to most relevant environmental predictors are shown in Figs. 4 and 5. All explanatory continuous variables suggested a curvilinear response at the spatial scale considered. Depth was the main environmental predictor explaining most of the variance with a percent contribution 45.3 (Table 2). Our results show that this species has a wide bathymetric range in our study area, but it is preferentially associated with depth range of 300–800 m (Fig. 4A). Slope was particularly important with a percent contribution 21.6. The probability of occurrence of this black coral species shows a curvilinear positive relationship with slope with higher probabilities in areas with high values of this terrain variable (Fig. 4B). The habitat preference of this black coral seems to be positively correlated with aspect (both directions; Fig. 4C,D), even if higher probabilities of finding *L. glaberrima* are positively related to high values of aspect EW. In particular, aspect EW had a higher percent contribution of 12.1 (Table 2) than the other direction (aspect NS percentage contribution of 4.9; Table 2). The probability of occurrence of this black coral has a negative curvilinear relationship with the kinetic energy due to currents at the seabed (Fig. 4E) suggesting that this species prefers environments characterized by moderate currents. This variable had a percent contribution of 8.1 (Table 2). Despite bottom temperature explains only a marginal part of model variance (percent contribution of 0.8; Table 2) the average response curve shows that higher probabilities of presence occur between 13 and 15 °C (Fig. 4F). The probability of *L. glaberrima* presence in relation to the habitat types resulted higher in few seabed categories as shown in Fig. 5: Deep-sea muddy sand, *Posidonia oceanica* beds, Facies of sandy muds with *Thenea muricata*, being the most relevant to the habitat selection of this black coral species, followed by Mediterranean biocoenosis of muddy detritic bottoms, Mediterranean biocoenosis of coastal terrigenous muds and Mediterranean communities of bathyal muds (Fig. 5). The percent contribution of this categorical variable was 7.3 (Table 2).

### Predicted distribution

**Figure 6:**
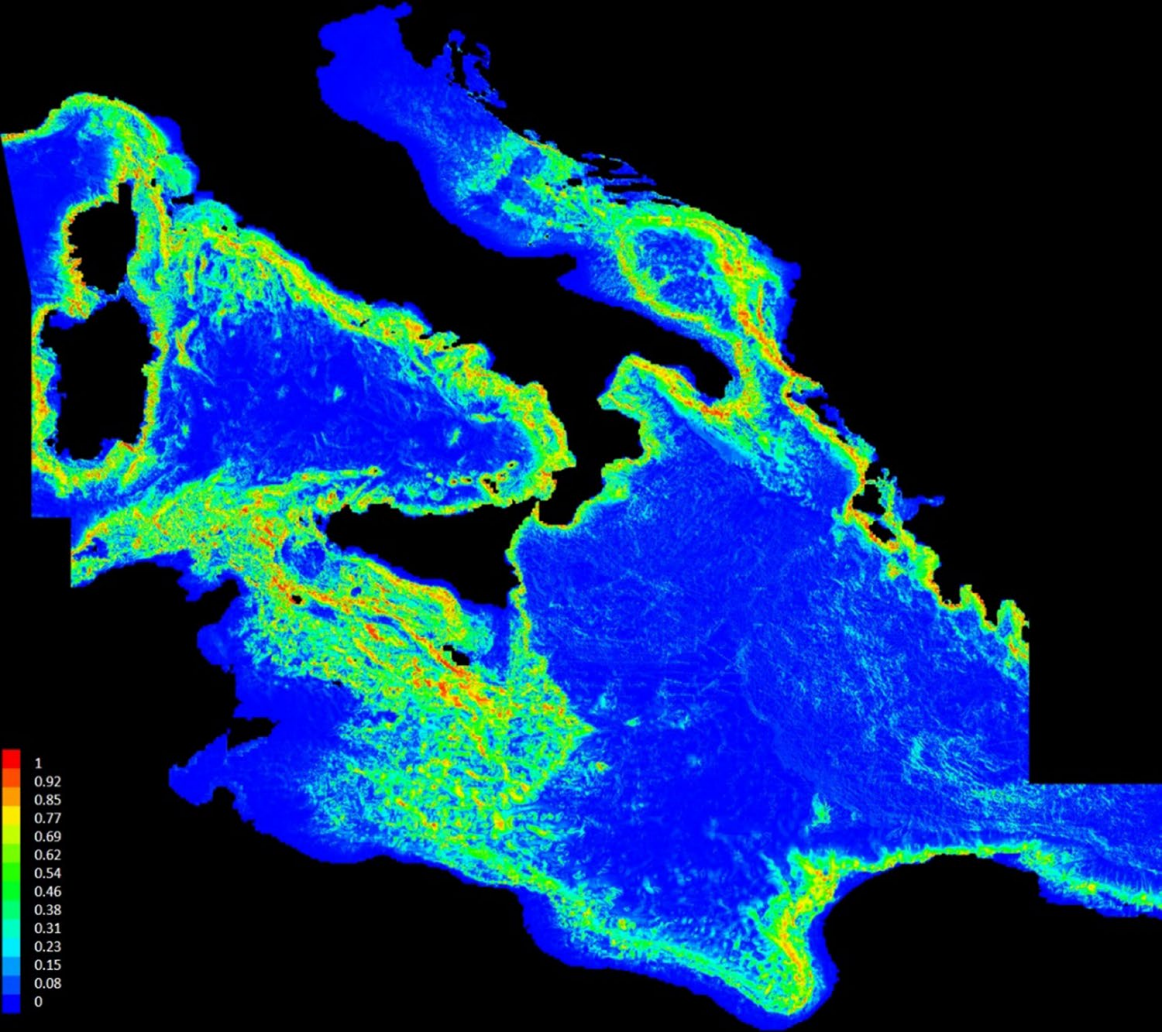
Spatial distribution map of *Leiopathes glaberrima* probabilities occurrence in the central sector of the Mediterranean Sea, as predicted from the MaxEnt modelling. Color ramp indicates the habitat suitability index for each cell, ranging from 0 to 1. This map was created with ArcGIS version 10.3 http://www.esriitalia.it by Tiziana Cillari.

Figure 6 shows the areas predicted to have suitable conditions for the occurrence of *L. glaberrima*, these were generally consistent with known presence areas. Predicted occurrences corresponded mainly to the modified Levantine Intermediate Waters of the Strait of Sicily, along the coasts of Tyrrhenian Sea, Ionian Sea, Sardinian Sea and Southern Adriatic Sea, where probabilities of species distribution were greater than 85% in small spotted areas. Consistent with ecological knowledge on the species, the model predicts *L. glaberrima* will occur most frequently on irregular elevated topographic structures associated to high values of slope (Fig. 2B). The map of the model standard deviation is showed in the Supplementary material (Figure S1).

## Discussion

This study enhances our understanding of the habitat requirements of a vulnerable deep-sea coral and provides the basis for the conservation of VMEs in the central sector of the Mediterranean Sea. Depth was an important environmental factor influencing the habitat preferences of this species in our study area (Table 2). The probability of occurrence of *L. glaberrima* was higher at depths between 300 and 800 m (Fig. 4A), despite this species has been recorded quite abundant also at shallower depths (200–600 m) in some regions of the Mediterranean Sea (i.e. in the Strait of Sicily and in the French canyons)^2,62^. *L. glaberrima* is known to have a wide bathymetric range (between 100 and 2048 m)^4^ but in the central Mediterranean it prefers depths around 600 m, similar results have been found in the Gulf of Mexico^10^. Still, we do not exclude that this species could also be found at deeper waters in our study area but because the limitation in the availability of data occurrence our model predictions might be biased toward this bathymetric range (Fig. 4A). Nevertheless, this preference for a well-defined depth range around 350–600 m has been observed for other CWC species (i.e*. L. pertusa* and *M. oculata*) in the Mediterranean Sea as these organisms are strongly influenced by the modified Levantine Intermediate Water mass^37,63^. In the Mediterranean the main CWC living grounds show a mosaic-like distribution where the maximum growth is known in the south western Adriatic, northern Ionian, Strait of Sicily, Gulf of Lion and around Sardinia canyons^37^. This spatial pattern corresponds to the belt of the modified LIW^6,37^ that support the growth of CWC and acts as a vector for larval dispersal thus connecting disjoint CWC provinces in the Mediterranean Sea.

Slope was another environmental predictor quite important for the habitat selection of *L. glaberrima* in our study area (Table 2), and the probability of presence of this black-coral species is positively related to this terrain variable (Fig. 4B). This preference for areas with gentle slope has been observed already around Malta in the Strait of Sicily^23^, therefore it likely that this environmental condition is an important prerequisite for the habitat suitability of *L. glaberrima*. In general, steep slope represents areas free of sediment and where hard rock outcrops can be exposed^64^, especially in canyons that are places with high sedimentation rate^65^. These hard substrate bottoms may be potential suitable habitats for CWC such as *M. oculata* and *L. pertusa*, as often observed in canyons^30,66^. Differently *L. glaberrima* seems to prefer a range of sea bottom types (i.e. from rocky bottoms to flat silted rocky bottoms^7^), but our results suggest higher occurrence in habitats characterised by soft sediments and gentle slope (Figs. 4B, 5). Mixed populations of black corals and other habitat-forming anthozoans can be found on the continental platform^14,67^ while only recently sparse colonies have been reported in bathyal habitats such as some French canyons^2^, probably in accordance with local current regimes^19^. For example in the canyons of the Gulf of Lion CWC are observed on different type of sediments from soft mud to hard rocks (and in some cases bauxite red mud) but mostly on steep slope^2^. In the Northwest Sicily tall colonies of *L. glaberrima* have been found on elevated rocky ridge (associated to high slope values) probably to avoid silting and exploit currents^19^. However in other cases black coral species (i.e. *Antipathella subpinnata*) showed high colony density on slight slopes (0° to 30°) while this was lower when the inclination increased^1^. Our results confirm this preference for gentle slope, but we do not exclude that these might be partially biased because the limited number of data points in our study area.

Since black corals are suspension feeders, their habitat selection is also forced by the hydrodynamic regime^1,13,14^. Our results confirm this as the environmental predictor aspect in both orientations North–South, East–West contributed to the habitat selection of *L. glaberrima* (Fig. 4C,D; Table 2). In general, this species favors quite energetic habitats to avoid silting and exploit the predominant current for feeding. Although zooplankton is the main prey in black corals diet^13^, particulate organic matter (POM) has been suggested also to be part of their diet^12^. Our study shows the preference of *L. glaberrima* for relatively exposed areas in the central sector of the Mediterranean Sea with moderate exposure to North–South direction, but higher probability of occurrence is also predicted in areas highly exposed to East–West direction (Fig. 4C,D). This supports previous findings on the distribution of this species at regional scale (South–West of Sardinia) where it was found in areas characterized by mesoscale cyclonic eddies^7^, but also in the Northwest of Sicily where a meadow of *L. glaberrima* was observed in the most exposed area of the Marco Bank^19^. In addition, our model output shows that the probability of occurrence of this black coral has a negative curvilinear relationship with the kinetic energy due to currents at the seabed (Fig. 4E), also confirming that this species prefers environments characterized by moderate currents. These results are in agreement with Bargain et al. (2018)^30^ that showed the importance of hydrodynamic factors (i.e. mean current velocity) as predictors of other CWC (i.e. *M. oculata* and *L. pertusa*) in the Cassidaigne canyon (eastern part of the Gulf of Lion), in fact currents are important for CWC, supplying food either directly or by creating sediment re-suspension, and preventing them from being buried^68,69^.

Although bottom temperature contributed very little to the overall habitat probability model (Table 2) still the results of the MaxEnt model evidenced a specie-specific optimum temperature range between 13 and 15 °C (Fig. 4F). However, we do not exclude that this result is probably biased by the fact that our occurrence points were limited to certain depths. Nevertheless, a specific preference for certain temperature ranges has been observed for some deep-sea coral species such as *L. pertusa* in the North Atlantic^70,71^ and *Isidella elongata* and *Funiculina quadrangularis* in the central Mediterranean Sea^40^.

Our final predictive map shows higher probability of habitat suitability along the Italian coasts mainly at the deep waters of the Strait of Sicily, along the coasts of Tyrrhenian Sea, Ionian Sea, Sardinian Sea and Southern Adriatic Sea, with some hot spots where the probability of finding *L. glaberrima* is greater than 85% (Fig. 6). This spatial pattern is likely to be linked to the stream of the modified LIW which has been suggested to support the growth and larval dispersal of CWC in the Mediterranean Sea^6,37^. The spatial pattern presented in Fig. 6 confirms the distribution of *L. glaberrima* in the coral provinces reported by Chimienti et al. 2019^6^ with hot spots in the Bari Canyon, Santa Maria di Leuca, South Malta and Sardinian Channel.

Modelling rare species is often problematic in terms of model overfitting and inaccuracy^72,73^. Still MaxEnt model has been suggested as a powerful tool to overcome this problem^74–76^ because this technique performs comparatively well in cases of low sample sizes^53,54^. This modelling approach has been applied for predictive CWC habitat mapping in several marine ecosystems in the Mediterranean and North Atlantic^10,25,29,55^, and in some cases performed better than other modelling techniques such as GLMs and ENFA^30^. However, a common limitation of habitat modelling studies is that model predictive maps should be validated using in situ surveys especially in cases of small sample size^77^. Still, this would require a post modelling underwater video surveys (with Remoted Operated Vehicles) implying elevated costs and time consuming. As the protection of Vulnerable Marine Ecosystems is becoming an urgent task to address worldwide, it is key to find a right compromise between research and conservation. In this study we acknowledge the fact that a limited number of occurrence points were used, however this limit is quite common for rare species such as *L. glaberrima* and for other deep-sea species that are difficult to sample (i.e. because of their deep bathymetric range therefore is not easy to collect information on its spatial distribution)^78^. To improve this study results a following step could be using a habitat modelling approach based on an ensemble of models such as Random Forest, GAMs, GLMs, including also other ecological predictors (e.g. curvature, pH, calcite saturation) as well as fishing effort (i.e. to identify potential conservation areas). Additional data points on *L. glaberrima* occurrence, especially at greater depths, would also enhance model performance and the final habitat distribution map. However, mapping rare species, even in cases of small sample size, represents an initial step for the protection of vulnerable species and associated marine ecosystems. For example, targeted ground truthing should be carried out in the areas that have been highlighted as distribution hot spots so that informed management decisions can be taken. If habitat suitability models are supported by surveys, management bodies should take appropriate conservation measures to minimize harmful impacts to these habitats from bottom fishing and other anthropogenic impacts. In this regard, both field observations and predictive maps derived from habitat suitability models should be use conjointly to improve the discovery and management of key important species such as *L. glaberrima* in the Mediterranean Sea.

## Conclusions

This study has important implications for the conservation of hard bottom coral gardens VMEs in the central sector of the Mediterranean Sea as it represents the first attempt to identify key areas at large scale (Fig. 5). Coral aggregations have now been internationally identified as special ecological features that need protection under the Convention of Biological Diversity^79^. On the other hand, the Food and Agriculture Organization (FAO) urged the establishment of MPAs where such VMEs are known to be or likely to occur in order to apply an ecosystem-based fishery management to deep-sea ecosystems^80^. In the Mediterranean Sea there are only few examples of Fishery Restricted Areas (FRAs) for the protection of VMEs (i.e. *Lophelia* Reefs of Santa Maria di Leuca, the area of cold hydrocarbon seeps off the Nile Delta, and the Eratosthenes Seamount) and some other relevant areas for the protection of sea pens and other corals have been highlighted in the Strait of Sicily and in the canyons in the Gulf of Lion^31,32,81^. Still the implementation of these FRAs is far from being in place. Although bottom trawling is forbidden from 1000 m depth onwards in the Mediterranean Sea, those deep-sea VMEs occurring at shallower depths which are completely unprotected. These include the coral gardens formed by *I. elongata*, *F. quadrangularis* and other habitat-forming organisms such black corals.

Sharp declines in shallow fisheries resources during the recent years^82,83^ occurred concurrently with the expansion of offshore fisheries to progressively greater depths^84,85^, threatening ecologically important and sensitive habitats such as canyons and seamounts^86^. For these reasons is urgent to adopt specific management and conservation measures that can protect deep sea habitats. Our study provides the first regional-scale map of the distribution of the suitable habitats of *L. glaberrima* representing the initial step for informing policy makers and guaranteeing that fishing activities are compatible with conservation plans and objectives.

## Supplementary Information

Supplementary Information.
