# Supplementary material for: Habitat suitability mapping of the black coral Leiopathes glaberrima to support conservation of vulnerable marine ecosystems
Source: Sci Rep. 2021 Aug 2;11:15661. doi: 10.1038/s41598-021-95256-4 (PMC8329192; doi:10.1038/s41598-021-95256-4)
Supplement: Supplementary file 1 — Supplementary Information. [file 41598_2021_95256_MOESM1_ESM.pdf]

Supplementary material

Table S1. Data occurrence of *Leiopathes glaberrima* reported in literature for the Central Mediterranean Sea. *from interviews with fishermen

Finding site	Region	Abundance	Gear	References
Greece	Adriatic Sea	colonies	ROV	1
Greece	Adriatic Sea	colonies	dredge	1
Montenegro	Adriatic Sea	colonies	ROV	2
Montenegro	Adriatic Sea	colonies	ROV	1
Vieste	Adriatic Sea	presence	long line *	3
Bari Canyon	Adriatic Sea	colonies	ROV	4
Cephalonia Island	Ionian Sea	colonies	ROV	5
Cephalonia ridge	Ionian Sea	colonies	long line	6
Southern Albanian Margin	Ionian Sea	colonies	trawl net	7
Cape Santa Maria di Leuca	Ionian Sea	presence	St. Andrew’s cross	8
Cape Santa Maria di Leuca	Ionian Sea	presence	long line	9
Cape Santa Maria di Leuca	Ionian Sea	presence	ROV	9
Cape Santa Maria di Leuca	Ionian Sea	presence	ROV	9
Cape Santa Maria di Leuca	Ionian Sea	colonies	St. Andrew’scross	10
Cape Santa Maria di Leuca	Ionian Sea	colonies	trawl net *	3
Santa Maria di Leuca	Ionian Sea	colonies	ROV	11
Santa Maria di Leuca	Ionian Sea	colonies	ROV	11
Porto Cesareo	Ionian Sea	colonies	long line *	3
Torre Inserraglio	Ionian Sea	presence	gill net*	3
Graham Shoal	Strait of Sicily	presence	ROV	12
Malta Escarpment	Strait of Sicily	colonies	ROV	13
Malta Graben	Strait of Sicily	forest	ROV	14
Southwestern Malta	Strait of Sicily	colony	trawl net	15
Favignana and Talbot Shoal	Strait of Sicily	presence	trawl net	15
Northern Levanzo Island	Tyrrhenian Sea	colony	trawl net	16
Cape San Vito	Tyrrhenian Sea	colonies	ROV	17
Marco Bank	Tyrrhenian Sea	colonies/forest	ROV	18
Marco Bank	Tyrrhenian Sea	forest	ROV	19
Aceste Seamount and Marco Bank	Tyrrhenian Sea	forest	ROV	4

Filicudi Island and adjacent Banks	Tyrrhenian Sea	colonies	ROV	17
Palmarola Island	Tyrrhenian Sea	colonies/forest	ROV	20
Vedove Shoal	Tyrrhenian Sea	colonies	ROV	18
Zannone Island	Tyrrhenian Sea	colonies/forest	ROV	20
Palinuro Seamount	Tyrrhenian Sea	colonies	ROV	21
off Ponza and Palmarola Islands	Tyrrhenian Sea	colonies/forest	ROV	4
off Ventotene	Tyrrhenian Sea	colonies	ROV	4
Montecristo Island	Tyrrhenian Sea	colonies	ROV	22
Northern edge of Skerki Bank	Sardinian Channel	colonies	trawl net	16
Rocky pinnacles off Carloforte	Sardinian Sea	colonies	ROV	23
Carloforte Shoal	Sardinian Sea	forest	ROV	24
Carloforte Shoal	Sardinian Sea	forest	ROV	25
Western Carloforte Island	Sardinian Sea	presence	ROV	26
Porto Canyon	Corsica Sea	presence	ROV	27
St. Lucia Bank	Ligurian Sea	colonies	ROV	18
Vado Ligure Canyon	Ligurian Sea	colonies	ROV	4

1. Taviani, M. *et al.* Reprint of ‘On and off the beaten track: Megafaunal sessile life and Adriatic cascading processess’. *Mar. Geol.* **375**, 146–160 (2016).

2. Angeletti, L. *et al.* New deep-water cnidarian sites in the southern Adriatic Sea. *Mediterr. Mar. Sci.* **15**, 263–273 (2014).

3. D’Onghia, G. *et al.* New records of cold-water coral sites and fish fauna characterization of a potential network existing in the Mediterranean Sea. *Mar. Ecol.* **37**, 1398–1422 (2016).

4. Chimienti, G., Bo, M., Taviani, M. & Mastrototaro, F. Coral Reefs of the World. in *Mediterranean Cold-Water Corals: Past; Present and Future* (eds. Orejas, C. & Jiménez C.) 213–243 (Springer, 2019).

5. Mytilineou, C. Chondromatidou, V. Smith, C.J. Papadopoulou, K. N. Cold water corals occurrence in the Eastern Ionian Sea using ROV. in *11th Panhellenic Symposium on Oceanography and Fisheries* (2015).

6. Mytilineou, C., Smith, C., Anastasopoulou, A., Papadopoulou, K. & Christidis, G. New cold-water coral occurrences in the Eastern Ionian Sea : Results from experimental long line fishing. *Deep. Res. Part I Oceanogr. Res. Pap.* **99**, 146–157 (2014).

7. Nasto, I. *et al.* Benthic invertebrates associated with subfossil cold water coral frames and hardgrounds in the Albanian deep waters (Adriatic Sea). *Turkish J. Zool.* **42**, 360-371 (2018).

8. Tursi, A., Mastrototaro, F., Matarrese, A., Maiorano, P. & D’Onghia, G. Biodiversity of the

white coral reefs in the Ionian Sea (Central Mediterranean). *Chem. Ecol.* **20**, 107–116 (2004).

9. Carlier, A. *et al.* Trophic relationships in a deep mediterranean cold-water coral bank (Santa Maria di Leuca, Ionian sea). *Mar. Ecol. Prog. Ser.* **397**, 125–137 (2009).

10. Mastrototaro, F. D'Onghia, G. *et al.* Biodiversity of the white coral and sponge community off Cape Santa Maria di Leuca (Mediterranean Sea). *Deep Sea Res. Part II Top. Stud. Oceanogr.* **57**, 412–430 (2010).

11. Capezzuto, F. *et al.* Cold-water coral communities in the Central Mediterranean: aspects on megafauna diversity, fishery resources and conservation perspectives. *Rend. Lincei* **29**, 589–597 (2018).

12. Greenpeace. *I tesori sommersi del Canale di Sicilia. No Trivelle Tour*. (2012).

13. Angeletti, L., Mecho, A., Doya, C., Micallef, A. & Huvenne, V. Georgiopoulou, A. Taviani, M. First report of live deep water cnidarian assemblages from the Malta Escarpment. *Ital. J. Zool.* **82**, 291–297 (2015).

14. Deidun, A. *et al.* First characterisation of a Leiopathes glaberrima (Cnidaria: Anthozoa: Antipatharia) forest in Maltese exploited fishing grounds. *Ital. J. Zool.* **82**, 271–280 (2015).

15. Massi, D. *et al.* Spatial distribution of the black coral Leiopathes glaberrima (Esper, 1788) (Antipatharia: Leiopathidae) in the Mediterranean: a prerequisite for protection of Vulnerable Marine Ecosystems (VMEs). *Eur. Zool. J.* **85**, 170–179 (2018).

16. Arena, P. & Li Greci, F. Indagine sulle condizioni faunistiche e sui rendimenti di pesca dei fondi batiali della Sicilia Occidentale e della bordura settentrionale dei banchi della soglia siculo-tunisina. *Quad. del Lab. di Tecnol. della Pesca* **1**, 157–201 (1973).

17. Bo, M. *et al.* Coral assemblages of the Calabrian Coast (South Italy) with new observations on living colonies of Antipathes dichotoma. *Ital. J. Zool.* **78**, 231–242 (2011).

18. Bo, M., Bava, S., Canese, S., Angiolillo, M. & Cattaneo-Vietti, R. Bavestrello, G. Fishing impact on deep Mediterranean rocky habitats as revealed by ROV investigation. *Biol. Conserv.* **171**, 167–176 (2014).

19. Bo, M. *et al.* The coral assemblages of an off-shore deep Mediterranean rocky bank (NW Sicily, Italy). *Mar. Ecol.* **35**, 332–342 (2014).

20. Ingrassia, M. *et al.* Black coral (Anthozoa, Antipatharia) forest near the western Pontine Islands (Tyrrhenian Sea). *Mar. Biodivers.* **46**, 285–290 (2016).

21. Bo, M. *et al.* Phylogenetic relationships of Mediterranean black corals (Cnidaria : Anthozoa : Hexacorallia) and implications for classification within the order Antipatharia. *Invetrebrate Syst.* **32**, 1102–1110 (2018).

22. Bo, M., Canese, S. & Bavestrello, G. Discovering Mediterranean black coral forests: Parantipathes larix (Anthozoa: Hexacorallia) in the Tuscan Archipelago, Italy. *Ital. J. Zool.* **81**, 112–125 (2014).

23. Cau, A. *et al.* Deepwater corals biodiversity along roche du large ecosystems with different habitat complexity along the south Sardinia continental margin (CW Mediterranean Sea).

Mar. Biol. **162**, 1865–1878 (2015).

24. Cau, A. *et al. Leiopathes glaberrima forest from South west Sardinia: A thousand years old nursery area for the small spotted catshark Scyliorinus canicula*. (2013).

25. Bo, M. *et al.* Persistence of pristine deep-sea coral gardens in the Mediterranean Sea (SW Sardinia). *PLoS One* **10**, 1–21 (2015).

26. Angiolillo, M. & Canese, S. Deep Gorgonians and Corals of the Mediterranean Sea. *Corals a Chang. World* (2018). doi:10.5772/intechopen.69686

27. Sartoretto, S. New records of Dendrobrachia bonsai (Octocorallia: Gorgonacea: Dendrobrachiidae) in the western Mediterranean Sea. *Mar. Biodivers. Rec.* **5**, 1–4 (2012).

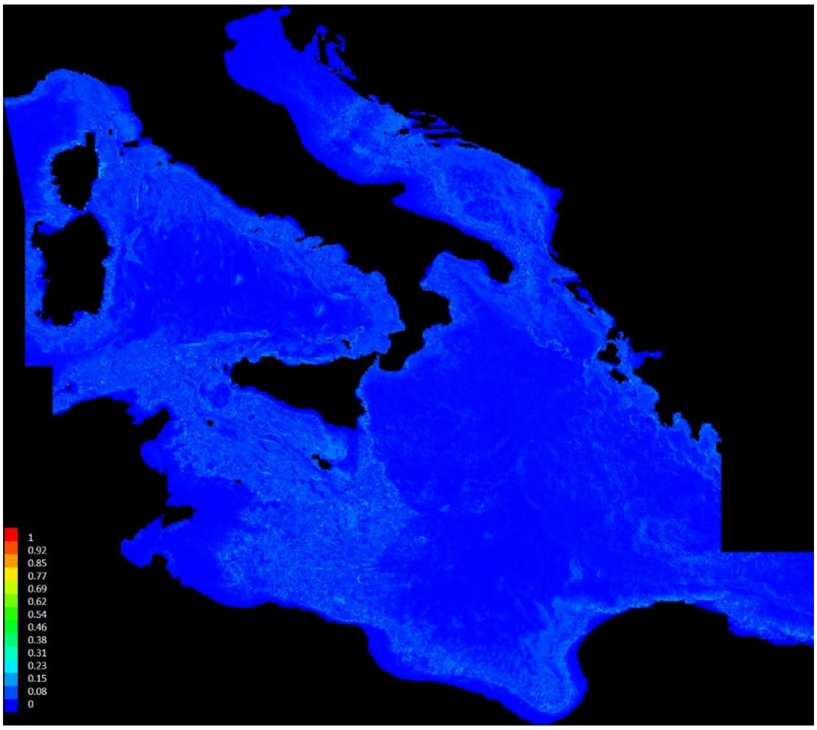

Figure S1. Standard deviation of spatial distribution of Leiopathes glaberrima in the central sector of the Mediterranean Sea, as predicted from the MaxEnt modelling. Color ramp indicates the standard deviation values. This map was created with ArcGIS version 10.3 http://www.esriitalia.it by Tiziana Cillari.
